# Procalcitonin Detection in Veterinary Species: Investigation of Commercial ELISA Kits

**DOI:** 10.3390/ani10091511

**Published:** 2020-08-26

**Authors:** Federica Battaglia, Valentina Meucci, Rosalba Tognetti, Francesca Bonelli, Micaela Sgorbini, George Lubas, Carlo Pretti, Luigi Intorre

**Affiliations:** Department of Veterinary Science, University of Pisa, 56122 Pisa, Italy; federica.battaglia@phd.unipi.it (F.B.); rosalba.tognetti@unipi.it (R.T.); francesca.bonelli@unipi.it (F.B.); micaela.sgorbini@unipi.it (M.S.); george.lubas@unipi.it (G.L.); carlo.pretti@unipi.it (C.P.); luigi.intorre@unipi.it (L.I.)

**Keywords:** procalcitonin, ELISA, equine, canine, validation

## Abstract

**Simple Summary:**

Among sepsis biomarkers, procalcitonin resulted to be a specific indicator of bacterial infection or severity of infection, and to be a good control of the success of a therapeutic procedure. The clinical studies on the relevance of procalcitonin as a sepsis predictor in veterinary patients are few, likely due to the total absence of validated assays. For this reason, this study aimed to investigate commercial ELISA kits for the detection of canine and equine procalcitonin. Validation was performed evaluating linearity, limits of detection (LOD), recovery, and intra-assay and inter-assay variability; furthermore, clinical samples were analyzed. The results of the present study demonstrate that the human PCT ELISA kit is suitable to detect equine procalcitonin with a LOD of 56 ng/mL, and the canine recombinant PCT ELISA kit can be used to measure canine procalcitonin in plasma samples, showing an intra-assay and inter-assay coefficient of variation less than 20% and a LOD of 11 pg/mL.

**Abstract:**

In human medicine, procalcitonin (PCT), the precursor of calcitonin, is used for the rapid identification of the origin and severity of sepsis. In veterinary medicine, PCT has been studied in horses, cattle, and dogs, but the use of PCT in diagnostic and/or prognostic settings is not possible because of the lack of validated assays to obtain reference ranges. The aim of the present study was the investigation of commercially available ELISA kits for the detection of canine and equine PCT in plasma samples. Validation of the ELISA kits was performed by using species-specific recombinant proteins spiked both in plasma and buffer samples; linearity, limit of detection (LOD), recovery, and intra-assay and inter-assay variability were calculated. Moreover, clinical samples obtained from sick and healthy animals were also analyzed with the tested kits. Canine PCT was measured with a recombinant canine and a canine PCT ELISA kit. Equine PCT was measured with an equine and a human ELISA PCT kit. Our data demonstrate that the canine recombinant PCT ELISA kit can be used to measure canine PCT in plasma samples, showing an intra-assay and inter-assay coefficient of variation less than 20% and a LOD of 11 pg/mL, whereas the present results do not support the use of the canine PCT ELISA kit. The human PCT ELISA kit is suitable to detect equine PCT with a LOD of 56 ng/mL, whereas the equine PCT ELISA kit did not detect recombinant equine PCT.

## 1. Introduction

Procalcitonin (PCT) is a precursor of the hormone calcitonin and consists of 116-amino acids, which are normally processed by a specific protease to calcitonin, katacalcin, and an N-terminal residue [1]. PCT is recognized as a highly specific and early marker for microbial infections and sepsis in human medicine [2]. PCT is markedly elevated within 2 to 4 h in severe forms of systemic inflammation or in bacterial infections, and this condition persists until recovery. Elevation of serum PCT in humans is used to distinguish patients with severe bacterial infections from those with severe non-septic conditions, to guide and reduce the antimicrobial treatment, and as a prognostic parameter [3]. For these reasons, PCT is very important to rapidly diagnose sepsis, minimize morbidity and mortality, and decrease unnecessary antibiotic use. Several methods are available to detect the PCT concentration in human blood, mostly using chemiluminescence [4,5], immunofluorescence [6], immunoluminometry [7,8], and also, surface plasmon resonance biosensors [9,10]. All of these methods rely on a combination of the same monoclonal mouse anti-katacalcin antibodies and monoclonal or polyclonal sheep anti-calcitonin antibodies (different among the assays). In addition, there are tests called “point-of-care testing”, which are rapid semi-quantitative immunochromatographic tests that provide a result within 30 min. The use of PCT in diagnostic and/or prognostic settings in veterinary species is not possible because of the lack of appropriate and validated analytical methods to measure PCT concentrations in plasma and to obtain reference ranges. Furthermore, the available assays are marketed for research use only and not for diagnostic purposes. Currently, PCT in veterinary species can be determined by using enzyme-linked immunosorbent assays (ELISA). Commercially available ELISA kits for veterinary species are often not validated in the target species, are expensive, and not applicable in clinical settings. Floras et al. [11] reported that one of the commercial ELISA kits measuring serum PCT level in dogs did not contain PCT in the standard calibrator, had intra-assay variability between 18.9% and 77.4%, and inter-assay variability between 56.1% and 79.5%, and therefore, was not suitable for PCT measurement in dogs. Only a kit targeted for canine PCT reported the use of recombinant canine PCT as standard calibrator (Biovendor Asheville, North Carolina, NC, USA). Goggs et al. [12] confirmed that the Biovendor canine PCT ELISA assay was able to detect canine PCT and can be used to determine plasma PCT concentrations in dogs. Furthermore, Easley et al. [13] reported the evaluation of PCT concentrations in dogs as a prognostic factor by using the same recombinant ELISA kit. Yilmaz et al. [14] reported the use of a commercial human ELISA kit for the detection of canine PCT but no validation data are reported. Equine PCT has been measured by using a commercial equine ELISA kit [15,16,17]. Rieger et al. [18] reported the development of a sandwich ELISA for the quantification of equine PCT in equine plasma samples based on monoclonal antibodies targeted against human PCT. Teschner et al. [19] used the method developed by Rieger et al. [18], reporting that PCT concentrations were quite high in horses that have colic due to endotoxemia with respect to control group (23,532.4 vs. 385.3 ng/mL); this method has been used by Barton et al. [20] to measure PCT in bronco alveolar fluid (BALF), showing that PCT level increased in horses with chronic pneumopathies and there was a correlation between plasma and BALF PCT. Pusterla et al. [21] evaluated PCT mRNA levels by real-time PCT, showing that there was not a significant difference between septic and non-septic foals.

The aim of this study was to evaluate commercially available ELISA kits targeted for dog and horse for the detection of their respective plasma PCT levels. The validation of the kits was performed by using species-specific recombinant PCT. PCT-spiked samples in buffer and in plasma were analyzed. Finally, a first approach on the use of the validated kits in clinical samples was performed.

## 2. Materials and Methods

### 2.1. Animals

Plasma samples from 5 heathy and 5 sick dogs were collected to measure PCT concentrations by using cPCT and rcPCT ELISA kits. Plasma from 5 healthy and 5 sick horses were collected to measure PCT concentrations by using ePCT and hPCT ELISA kits. Details concerning animals’ signalment were reported in Table 1. The following data were recorded for both healthy and sick animals, in order to include dogs and horses in the “healthy” or in the “Systemic Inflammatory Response Syndrome (SIRS)” group: presence of abnormal leukocyte count or distribution (leukopenia, leukocytosis, or >10% band neutrophils), hyperthermia or hypothermia, tachycardia, or tachypnea [22,23]. Moreover, the presence of a final diagnosis of inflammation due to bacteria was also considered mandatory. All the 10 healthy dogs and horses enrolled as controls showed no signs of SIRS, thus, they were included in the healthy group. Dogs and horses presenting with 2 or more criteria were considered affected by SIRS. Despite the fact that the PCT evaluation in canine and equine species used the SIRS score for the inclusion of sick animals, the specificity of those criteria may be low. Thus, our SIRS group was composed of dogs (*n* = 5) and horses (*n* = 5) with a positive SIRS score (≥2 criteria) plus diagnosis of bacterial infection in order to only include animals truly affected by SIRS due to bacteria (Table 2).

Blood samples for PCT concentrations were collected from the jugular vein using a sterile syringe into a 2.5 mL heparinized tube. Samples were immediately centrifuged at 3000 rpm for 10 min. All plasma samples, from horses and dogs with and without sepsis, were then divided in 4 aliquots and stored at −80 °C from the time of collection and thawed just before use. There was no intravenous administration of calcium before blood collection, so as to not influence plasma PCT concentrations. The present study was approved by the Institutional Animal Care and Use Committee of the University of Pisa (Prot. N° 2825/14). An owner’s written consent was obtained for plasma collection.

### 2.2. PCT ELISA Kit

All investigated ELISA kits used in this study are sandwich immunoassays. Canine PCT was measured with two different commercial ELISA kits: the recombinant canine procalcitonin ELISA kit (rcPCT, Biovendor Asheville, North Carolina, NC, USA) and the canine procalcitonin ELISA kit (cPCT, TSZ ELISA, Framingham, MA, USA). Equine PCT was measured with two different commercial ELISA kits: the equine procalcitonin ELISA kit (ePCT, Mybiosource, San Diego, CA, USA) and the human procalcitonin ELISA kit (hPCT, Sigma-Aldrich, Milan, Italy). The preparation of reagents and all incubations and washes were performed according to the manufacturer’s instructions. The optical density (OD) of the samples was determined with a microplate reader set to a wavelength of 450 nm for all kits except for the rcPCT ELISA kit. In this case, the absorbance was determined subtracting the readings at 630 nm from the readings at 450 nm. The OD of the blank well was subtracted from each sample OD. The standard curve was constructed by plotting the mean absorbance of standards against the known concentration of standards for all ELISA kits except for the rcPCT ELISA kit. In this case, the concentration was expressed in logarithmic scale. This was subsequently used to determine the PCT concentration in samples. Samples with absorbances exceeding the absorbance of the highest standard have been measured with higher dilution. The final concentration of samples calculated from the standard curve has been multiplied by the respective dilution factor. The validation parameters (LOD; Calibration Range; Intra-assay and Inter-assay variation) of the tested kits supplied by the manufacturer are reported in Table S1.

### 2.3. Validation of ELISA Kit

The ELISA kits were validated evaluating linearity, limits of detection (LOD), limits of quantitation (LOQ), limits of blank (LOB), recovery, and intra-assay and inter-assay variability. Three calibration curves based on the analysis of duplicate standard solutions supplied by the manufacturer were constructed for each PCT ELISA kit. Recombinant canine PCT (Biovendor, Asheville, NC, USA) was reconstituted in water and dilutions of 12.5, 25, 50, 100, 200, 400, and 800 pg/mL were used to construct calibration curves for both the cPCT and rcPCT ELISA kit (*n* = 3). Recombinant equine PCT (Biovendor, Asheville, North Carolina, NC, USA) was reconstituted in water and dilutions of 100, 500, 1000, 5000, 10,000, and 25,000 pg/mL were used to construct calibration curves for both the ePCT and hPCT ELISA kit (*n* = 3). Moreover, the following dilutions of recombinant equine PCT were tested in hPCT ELISA kit: 500, 1000, 5000, 10,000, and 25,000 ng/mL. LOD has been calculated as the concentration of PCT giving absorbance higher than the mean absorbance of the blank (buffer) plus three standard deviations of the absorbance of the blank: A blank + 3 × SD blank. LOQ has been calculated as concentration of PCT giving absorbance higher than the mean absorbance of the blank (buffer) plus ten standard deviations of the absorbance of the blank: A blank + 10 × SD blank. LOB has been calculated as concentration of PCT giving absorbance higher than the mean absorbance of the blank (buffer) plus 1.645 standard deviations of the absorbance of the blank: A blank + 1.645 × SD blank. Canine and equine plasma samples obtained from healthy subjects were pooled and spiked with recombinant proteins at 100, 500, and 1000 pg/mL and used to obtain validation parameters for cPCT, rcPCT, and ePCT ELISA kits. Equine plasma samples were also spiked with recombinant equine PCT protein at 0.1, 0.5, 1, 1000, 5000, and 10,000 ng/mL and were used to obtain validation parameters for the hPCT ELISA kit. Plasma spiked samples were analyzed in 5 replicates and the mean, standard deviation (SD), coefficient of variation (CV), and recovery were calculated. Intra-assay CV was calculated for each plate and for each measured concentration. Inter-assay CV was determined on three plates at each concentration. Acceptance criteria for CV were set within 20% of the nominal value. Cross-reactivity was calculated by comparing standard curves of human recombinant PCT and equine recombinant PCT obtained in the hPCT ELISA kit by using the formula: EC50 human PCT/EC50 equine recombinant PCT. EC50 is defined as the concentration of PCT corresponding to 50% of the maximum OD in the calibration curve.

### 2.4. Statistical Analysis

Data analysis was performed using Graph Pad Prism v. 8 software (GraphPad Software, San Diego, CA, USA) for Mac. The Kolmogorov–Smirnov test was performed to evaluate data distribution. The Mann–Whitney test was used to evaluate differences between healthy and sick animals. The Spearman test was used to compare results obtained with different ELISA kits. A level of *p* < 0.05 was considered as a limit of statistical significance.

## 3. Results

### 3.1. Validation of ELISA Kit

The validation parameters of the rcPCT ELISA kit obtained in the plasma samples spiked with recombinant canine PCT are reported in Table 3.

The cPCT ELISA kit did not detect recombinant canine PCT at any assayed concentrations in both the buffer and spiked plasma samples (OD < LOD) and it could not be validated by using a recombinant protein. Analysis of the dilutions of the calibration standard supplied in the cPCT ELISA kit generated standard curves with a mean r^2^ of 0.9432 (*n* = 3) (Figure S1). Analysis of the dilutions of the calibration standard supplied in the rcPCT ELISA kit generated standard curves with a mean r^2^ of 0.9814 (*n* = 3). The recombinant canine PCT not supplied with the kit and reconstituted in water showed corresponding results (mean r^2^ = 0.9820) (Figure S2). The ePCT ELISA kit did not detect recombinant equine PCT at any assayed concentrations in both water and spiked plasma samples (Tables S2 and S3). Recombinant equine PCT dilutions in water ranging from 100 to 25,000 pg/mL were not detected by hPCT ELISA kit. Analysis of the dilutions in water ranging from 500 to 25,000 ng/mL generated standard curves with a mean r^2^ of 0.9200 (Figure S3). The validation parameters of the hPCT ELISA kit obtained in the plasma samples spiked with recombinant equine PCT are reported in Table 4.

A cross reactivity of 0.02% was obtained for equine recombinant PCT measured by the hPCT ELISA kit. Analysis of the dilutions of the calibration standard supplied in the ePCT ELISA kit generated standard curves with a mean r^2^ of 0.9904 (*n* = 3) (Figure S4). Analysis of the dilutions of the calibration standard supplied in the hPCT ELISA kit generated standard curves with a mean r^2^ of 0.9470 (*n* = 3) (Figure S5).

### 3.2. Analysis of Plasma Samples

#### 3.2.1. Canine Plasma

The plasma PCT concentrations measured by using the cPCT and rcPCT ELISA kits in the samples of healthy and sick dogs are reported in Table 5.

**Figure 1:**
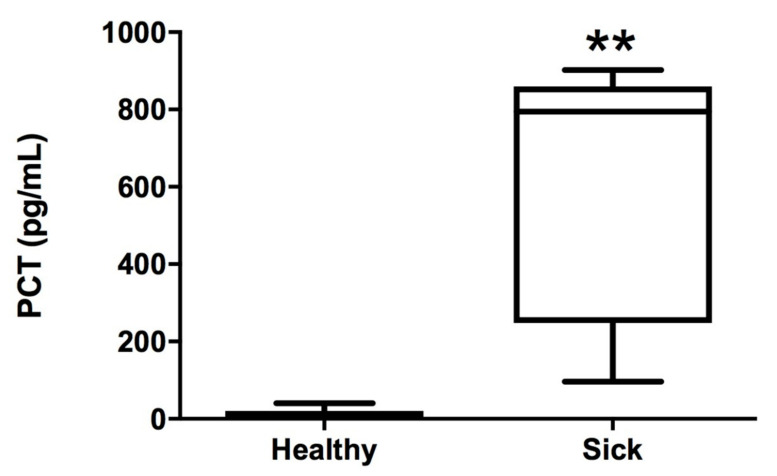
Concentrations of PCT measured by the rcPCT kit in healthy and sick canine plasma samples (** *p* < 0.01).

No correlation was found between the two assayed kits (Spearman r = 0.1161; *p* = 0.7494). Plasma PCT concentrations measured by the rcPCT ELISA kit were statistically higher in sick (median 725.2 pg/mL, range 79.3–944.7 pg/mL) than in healthy dogs (median 0, range < LOD—61.60 pg/mL) (*p* < 0.03) (Figure 1), whereas no statistically significant differences were observed for the cPCT ELISA kit between healthy (median 22.5 pg/mL, range < LOD—1178.0 pg/mL) and sick dogs (median 0, range < LOD—783.0 pg/mL) (*p* > 0.05).

#### 3.2.2. Equine Plasma

The plasma PCT concentrations measured by using the ePCT and hPCT ELISA kits in the samples of healthy and sick horses are reported in Table 6.

**Figure 2:**
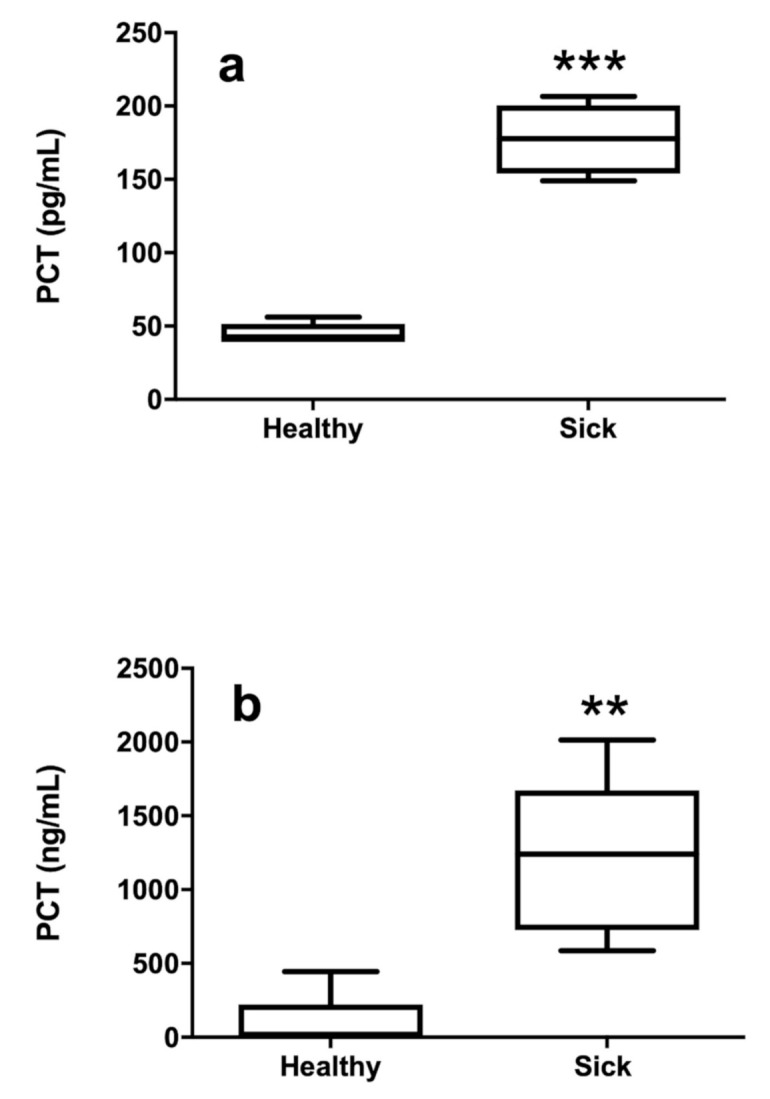
Concentrations of PCT measured by the ePCT (**a**) and hPCT (**b**) kits in healthy and sick equine plasma samples (** *p* < 0.01; *** *p* < 0.001).

A statistically significant correlation was found between the two assayed kits (Spearman r = 0.6868; *p* = 0.0283). Plasma PCT concentrations measured by the ePCT ELISA kit were statistically higher in sick (median 177.8, range 149.1–206.5 pg/mL) than in healthy horses (median 42.7, range 39.3–56.2 pg/mL) (*p* < 0.009) (Figure 2a). Plasma PCT concentrations measured by the hPCT ELISA kit were statistically higher in sick (median 1101.0 pg/mL, range 538.6–2084.0 pg/mL) than in healthy horses (median 0, range < LOD—67.9 pg/mL) (*p* < 0.04) (Figure 2b).

## 4. Discussion

**Figure 3:**
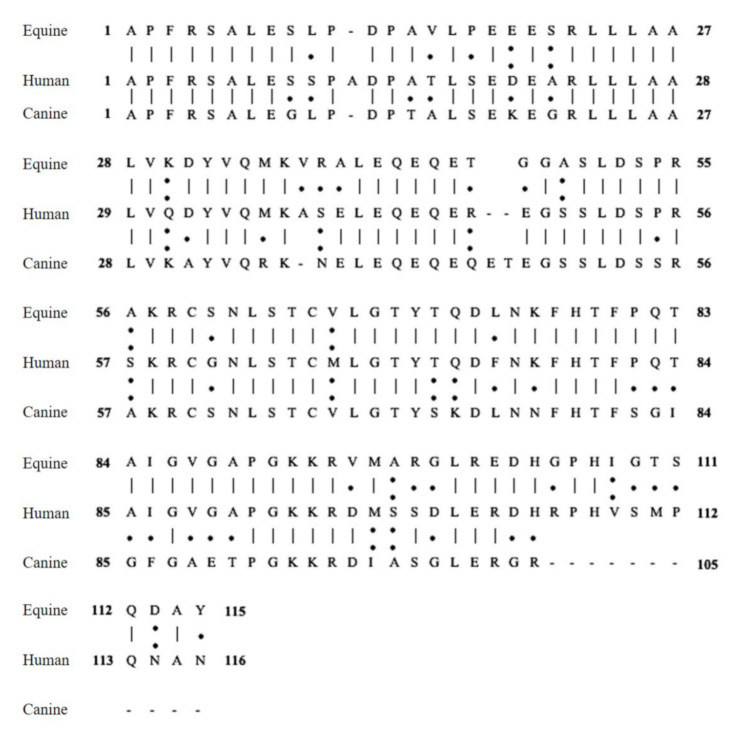
Alignment of the amino acid sequences of equine–human–canine procalcitonin. Numbers indicate amino acids. The symbols between the amino acid sequences represent conserved sequences (|), conservative mutations that are different amino acids with similar biochemistry proprieties such as hydrophobicity, size and charge (:), semi-conservative mutations (·), and gaps (-).

The present study is a first investigation regarding the validation of commercially available ELISA kits used to detect PCT in dogs and horses. Validation data are extremely useful to allow the kits to be used to obtain the reference ranges. The immunochemical quantification of PCT in human medicine is state of the art for sepsis diagnosis, course control of the disease, and antibiotic stewardship. The same use has been proposed for this sepsis biomarker in veterinary medicine. Although several ELISA kits are commercially available, the use of PCT in diagnosis and/or prognosis settings is not possible because of the lack of species-specific reference ranges and validated assays. The present study showed that the cPCT ELISA kit used was not able to detect and measure canine recombinant PCT either in buffer or plasma samples. Different dilutions of recombinant canine PCT showed OD lower than the blank samples, confirming a lack of binding of PCT to the kit antibodies. The cPCT ELISA kit could not be validated. The cPCT ELISA also generated inconsistent results for canine plasma of both healthy and septic dogs. These results do not support the use of this commercial ELISA for the detection of PCT in dogs. Dog PCT has a homology within the amino acid sequence to the human PCT of 67% [1,24] (Figure 3).

The results regarding canine the rcPCT ELISA kit are in agreement with the study of Goggs and collaborators [12], confirming that this assay can be used to measure plasma PCT concentrations in dogs and that dogs with sepsis have increased concentrations of PCT compared with healthy controls. The rcPCT ELISA kit has been validated, showing CVs for spiked concentrations in blank plasma all within the acceptance criteria and very good values of recovery. Intra-assay, inter-assay, and recoveries values obtained in this study are similar to the values reported by the assay manufacturer (intra-assay CV 3.7–4.6%, inter-assay CV 6.7–7.5%, recovery 88.0–99.0%). Goggs et al. [12] reported only intra-assay values which were also similar to the values obtained in this study (1.8–4.5%). The obtained LOD is higher than those reported by the manufacturer (3.6 pg/mL) and by Goggs et al. [12] (3.4 pg/mL), and this can be due to the use of water as the recombinant protein diluent respect to the sample diluent of the ELISA kit. Further studies are needed to establish reference ranges in dogs by evaluating the influence of age, gender, and breed prior the clinical implementation of PCT.

Human PCT consists of 116 amino acids with 13kDa molecular weight [25]. Equine PCT consist of 115 amino acids with a molecular weight of 12.5 kDa. Equine PCT has a homology within the amino acid sequence to the human PCT of 74% [26] (Figure 3). In agreement with this analogy of amino acid sequence between horse and human, the recombinant equine PCT protein, used as a positive control, was detected by the hPCT ELISA kit. The same dilutions of recombinant equine PCT were not detected with the ePCT ELISA kit. This result could be related to the recombinant version of PCT used as a positive control. Recombinant protein was not in its native form; conversely, the supplied protein standard of the ePCT ELISA kit was in its native form. The recombinant protein may have been slightly different from the target used to raise the capture antibody, which could have interfered with immunodetection. Furthermore, the buffer used to yield some dilutions may have interfered with the assay. The buffer may have impacted the stability of the recombinant protein or impaired the interaction with the capture antibody. Although the same dilution series of recombinant equine PCT analyzed with the hPCT ELISA kit was not detected with the ePCT ELISA kit, the present study confirmed that the commercial ePCT ELISA kit detects equine PCT in plasma sample, showing a statistically significant difference between healthy and sick horses. A limitation of the study is the small sample size of clinical samples. The concentration of PCT in the equine plasma samples obtained in this study with the use of the hPCT ELISA kit and recombinant equine PCT as calibrators is similar to those reported by Rieger et al. [18], who used an equine recombinant PCT as standard and human antibodies. Rieger et al. [18] showed a higher cross-reactivity with respect to the present study. Furthermore, PCT concentrations in the plasma samples obtained by using the hPCT ELISA kit and the recombinant equine PCT as standard calibrators are higher than those reported by using the ePCT ELISA kit both in this and other studies [15,16,17]. This could be related to the use of human antibodies used to detect equine PCT. Validation data and equine plasma sample analysis suggest the use of the hPCT ELISA kit to detect equine PCT. However, the obtained recovery values of the hPCT kit in measuring equine PCT are much lower than the acceptable range of 85–115% for most assays. Further studies and a larger sample size are needed to allow the use of this kit with diagnostic purposes. Finally, the present study suggests the need of future research regrading reference ranges of both canine and equine PCT levels in healthy subjects.

## 5. Conclusions

The present study investigated commercial ELISA kits for the quantitative determination of PCT in dogs and horses. Based on this study, canine PCT can be measured in plasma by using the canine recombinant PCT ELISA kit (Biovendor Asheville, NC, USA). The human PCT ELISA kit (Sigma-Aldrich, Milan, Italy) is suitable to detect PCT concentrations in equine plasma samples, whereas the equine PCT ELISA kit (Mybiosource, San Diego, CA, USA) used did not detect recombinant standard protein and cannot be fully validated.

## Figures and Tables

**Table 1:** Details about signalment for the 20 animals studied (5 healthy and 5 sick dogs; 5 healthy and 5 sick horses). Legend: H—healthy; S—sick; F—female; M—male; MN—male neutered; G—gelding; S—stallion.

Animals	Signalment		
	Breed	Age (years)	Sex
H-dog-1	Labrador Retriever	6	M
H-dog-2	Labrador Retriever	2	F
H-dog-3	Mixed Breed	3	F
H-dog-4	Mixed Breed	5	MN
H-dog-5	Australian Shepherd	3	MN
S-dog-1	Maremmano Abruzzese Shepherd	8	F
S-dog-2	Mixed Breed	4	F
S-dog-3	Mixed Breed	6	M
S-dog-4	Border Collie	6	F
S-dog-5	Labrador Retriever	3	M
H-horse-1	Standardbred	10	F
H-horse-2	Standardbred	9	F
H-horse-3	Standardbred	5	F
H-horse-4	Arabian Horse	15	S
H-horse-5	Draft Horse	13	G
S-horse-1	Standardbred	6	F
S-horse-2	Thoroughbred	4	F
S-horse-3	Quarter Horse	7	F
S-horse-4	Quarter Horse	5	G
S-horse-5	Thoroughbred	4	S

**Table 2:** Clinical diagnoses and SIRS criteria which lead to a diagnosis of inflammation due to bacterial infection for the 10 sick animals included (5 dogs; 5 horses). Legend: S—sick; SIRS—systemic inflammatory response syndrome.

Animals	Diagnosis	SIRS Criteria Met	Details Concerning SIRS Criteria/Clinical Signs
S-dog-1	Pyometra	4 out of 4	All clinical signs present
S-dog-2	Septic peritonitis	3 out of 4	Alteration of WBC count, body temperature and heart rate
S-dog-3	Septic pleuritis	4 out of 4	All clinical signs present
S-dog-4	Pyometra	4 out of 4	All clinical signs present
S-dog-5	Pyoderma	3 out of 4	Alteration of WBC count, body temperature and heart rate
S-horse-1	Septic pleuritis and pneumonia	4 out of 4	All clinical signs present
S-horse-2	Septic pleuritis and pneumonia	4 out of 4	All clinical signs present
S-horse-3	Colic due to strangulating lesions	4 out of 4	All clinical signs present
S-horse-4	Colic due to strangulating lesions	4 out of 4	All clinical signs present
S-horse-5	Septic peritonitis	3 out of 4	Alteration of WBC count, body temperature and heart rate

**Table 3:** Validation parameters of the rcPCT ELISA kit obtained in the plasma samples spiked with recombinant canine PCT. CV—coefficient of variation; LOD *—limit of detection obtained in buffer spiked with recombinant canine PCT; LOQ *—limit of quantitation obtained in buffer spiked with recombinant canine PCT; LOB *—limit of blank obtained in buffer spiked with recombinant canine PCT.

Recombinant Canine PCT Concentration in Plasma (pg/mL)	Measured Canine PCT Concentration (pg/mL)	Recovery (%)	Intra-Assay CV (%)	Inter-Assay CV (%)	LOD * (pg/mL)	LOQ * (pg/mL)	LOB * (pg/mL)
100	97.2 ± 8.3	97.2	7.9	9.0	11	20	13
500	482.0 ± 19.2	96.4	5.6	2.3			
1000	1008.0 ± 116.5	116.5	10.8	12.4			

**Table 4:** Validation parameters of the hPCT ELISA kit obtained in the plasma samples spiked with recombinant equine PCT. CV—coefficient of variation; LOD *—limit of detection obtained in buffer spiked with recombinant equine PCT; LOQ *—limit of quantitation obtained in buffer spiked with recombinant canine PCT; LOB *—limit of blank obtained in buffer spiked with recombinant canine PCT.

Recombinant Equine PCT Concentration in Plasma (ng/mL)	Measured Equine PCT Concentration (ng/mL)	Recovery (%)	Intra-Assay CV (%)	Inter-Assay CV (%)	LOD * (ng/mL)	LOD * (ng/mL)	LOD * (ng/mL)
1000	680.0 ± 55.0	68.0	6.1	10.1	56	113	69
5000	3792.9 ± 107.0	75.0	2.3	6.6			
10,000	4797.7 ± 667.1	47.0	5.0	6.0			

**Table 5:** PCT concentrations in healthy and sick dogs measured with the cPCT and rcPCT ELISA kits. CV—coefficient of variation; LOD—limit of detection.

Sample	cPCT Kit		rcPCT Kit	
	Measured PCT Concentration (pg/mL, Mean ± SD)	CV%	Measured PCT Concentration (pg/mL, Mean ± SD)	CV%
H-dog-1	207.5 ± 293.4	141.4	<LOD	---
H-dog-2	<LOD	---	<LOD	---
H-dog-3	855.5 ± 456.1	53.3	<LOD	---
H-dog-4	<LOD	---	<LOD	---
H-dog-5	104.0 ± 83.4	80.2	40.0 ± 30.5	76.1
S-dog-1	453.0 ± 113.1	25.0	902.3 ± 59.9	6.6
S-dog-2	391.5 ± 553.7	141.4	794.2 ± 62.6	7.9
S-dog-3	<LOD	---	816.9 ± 164.6	20.1
S-dog-4	12.5 ± 17.68	142.0	399.4 ± 132.4	33.1
S-dog-5	<LOD	---	96.4 ± 24.1	25.0

**Table 6:** PCT concentrations in healthy and sick horses measured with the ePCT and hPCT ELISA kits. CV—coefficient of variation; LOD—limit of detection.

Sample	ePCT Kit		hPCT Kit	
	Measured PCT Concentration (pg/mL, Mean ± SD)	CV %	Measured PCT Concentration (pg/mL, Mean ± SD)	CV %
H-horse-1	46.9 ± 49.0	104.3	<LOD	---
H-horse-2	39.3 ± 38.2	97.2	445.0 ± 331.2	74.4
H-horse-3	42.7 ± 7.2	16.8	<LOD	---
H-horse-4	39.3 ± 23.9	60.7	<LOD	---
H-horse-5	56.2 ± 55.0	97.7	<LOD	---
S-horse-1	194.7 ± 64.5	33.1	1241.0 ± 0.0	0
S-horse-2	177.8 ± 28.7	16.1	1330.0 ± 298.1	28.9
S-horse-3	206.5 ± 62.1	30.0	865.1 ± 134.5	15.5
S-horse-4	159.2 ± 33.4	21.0	585.5 ± 66.2	11.3
S-horse-5	149.1 ± 19.1	12.8	2014.0 ± 99.4	5.0

## Supplementary Materials

The following are available online at https://www.mdpi.com/2076-2615/10/9/1511/s1, Figure S1: standard curves for cPCT ELISA kit, Figure S2: standard curves for rcPCT ELISA kit, Figure S3: standard curves for hPCT ELISA kit obtained with equine recombinant PCT, Figure S4: standard curves for ePCT ELISA kit, Figure S5: standard curves for hPCT ELISA kit, Table S1: performance characteristics of all analyzed kits supplied by the manufacturer, Table S2: buffer samples spiked with equine recombinant PCT and measured with ePCT ELISA kit, Table S3: equine plasma samples spiked with equine recombinant PCT and measured with ePCT ELISA kit.
