# Supplementary material for: Procalcitonin Detection in Veterinary Species: Investigation of Commercial ELISA Kits
Source: Animals (Basel). 2020 Aug 26;10(9):1511. doi: 10.3390/ani10091511 (PMC7552142; doi:10.3390/ani10091511)
Supplement: Supplementary file 1 [file animals-10-01511-s001.pdf]

Kit	Type of sample suitable for analysis	Calibration range	Intra- and inter-assay CV	LOD	LOD*	Calibration range*	Kit manufacturer
**cPCT**	Serum, plasma, cell culture supernatants, other biological fluids.	62.50-1000.00 pg/ml	NR	NR	---		TSZ ELISA
**rcPCT**	Serum, urine.	12.50-800.00 pg/ml	4.15% - 7.10%	3.60 pg/ml	11 pg/ml	12.5-800 pg/ml	Biovendor
**ePCT**	Serum, plasma, tissue homogenates, secretions, other body fluids, feces.	50.00-1600.00 pg/ml	<15.00% - <15.00%	10.00 pg/ml	---		Mybiosource
**hPCT**	Serum, plasma, cell culture supernatants.	27.43-20000.00 pg/ml	<10.00% - <12.00%	30.00 pg/ml	56 ng/ml	500-25.000 ng/ml	Sigma Aldrich

Table S1. Performance characteristics of all analyzed kits supplied by the manufacturer. CV: coefficient of variation; LOD: limit of detection; NR: data not reported by manufacturer.* Data obtained in the present study by using canine and equine recombinant PCT as standard calibrators.

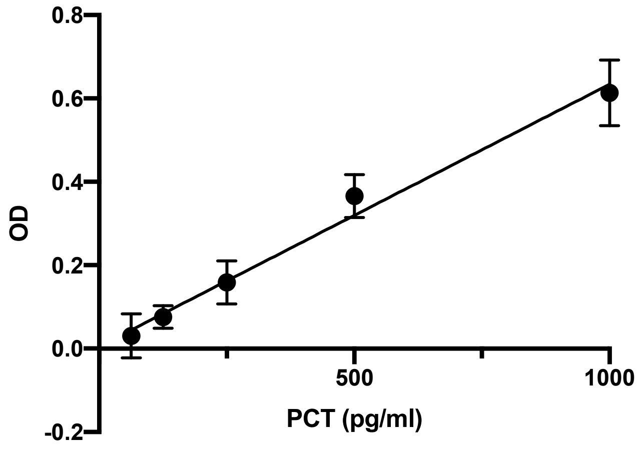

Figure S1. Standard curves for cPCT ELISA kit (n = 3); OD optical absorbance.

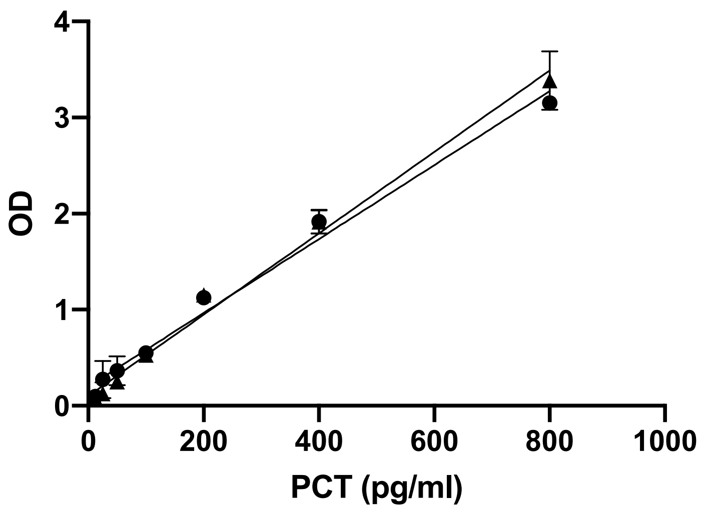

Figure S2. Standard curves for rcPCT ELISA kit (n = 3); OD optical absorbance. circles rcPCT supplied with the kit; triangles rcPCT not supplied with the kit and reconstituted in water.

Equine recombinant PCT concentration in water (pg/ml)	Mean OD	Measured Equine recombinant PCT concentration (pg/ml)
**0**	0.041	<LOD
**100**	0.628	903.9
**500**	0.615	884.9
**1000**	0.658	950.4
**5000**	0.674	974.0
**10000**	0.622	895.5
**25000**	0.646	931.3

Table S2. Buffer samples spiked with different amounts of equine recombinant PCT and measured with ePCT ELISA kit. LOD*, limit of detection; OD optical absorbance.

Equine recombinant PCT concentration in plasma (pg/ml)	Mean OD	Measured Equine recombinant PCT concentration (pg/ml)
**0**	0.355	488.6
**100**	0.357	490.9
**500**	0.320	435.2
**1000**	0.368	507.7

Table S3. Equine plasma samples spiked with different amounts of equine recombinant PCT and measured with ePCT ELISA kit. LOD*, OD optical absorbance.

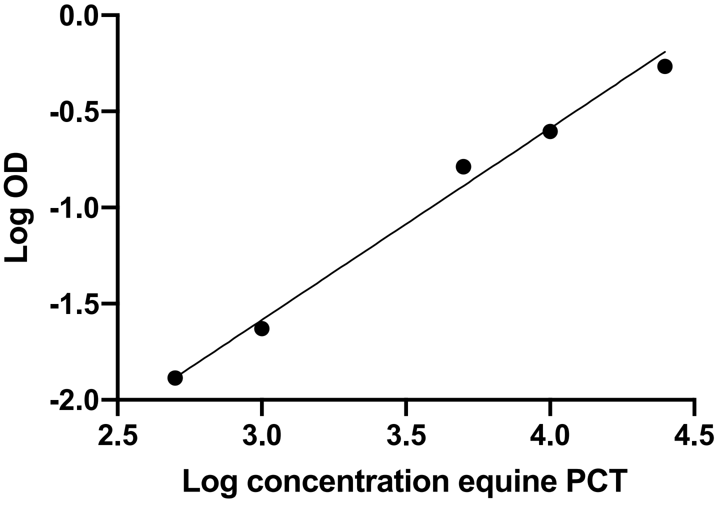

Figure S3. Standard curves for hPCT ELISA kit (n = 3) obtained with buffer spiked with different amount of equine recombinant PCT; OD optical absorbance.

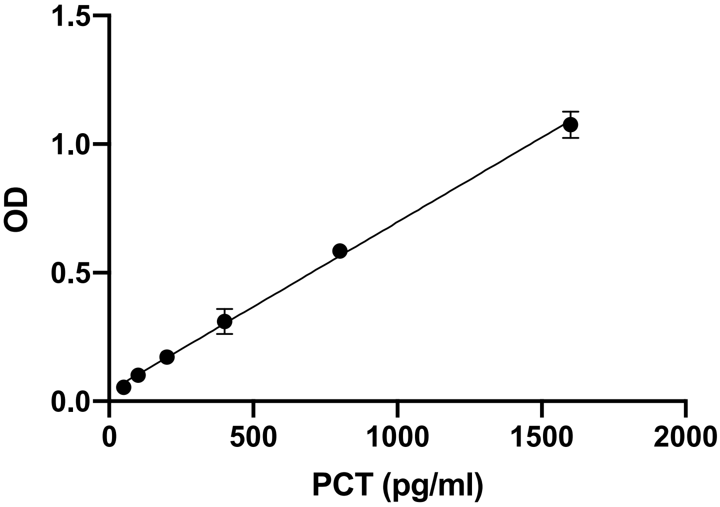

Figure S4. Standard curves for ePCT ELISA kit (n = 3); OD optical absorbance.

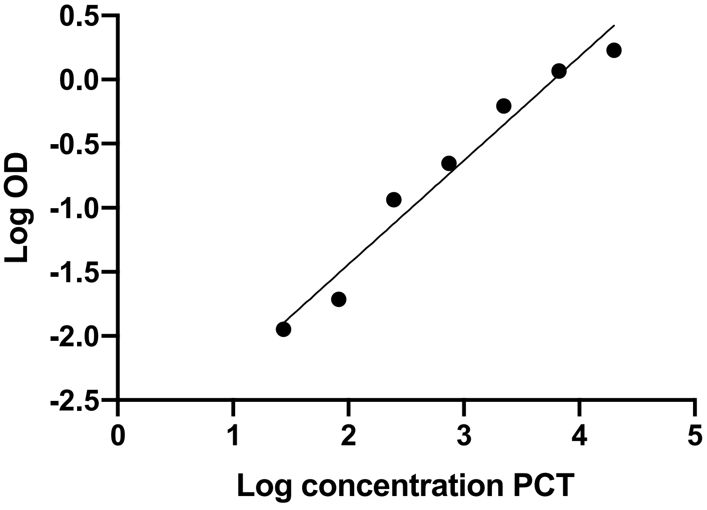

Figure S5. Standard curves for hPCT ELISA kit (n = 3); OD optical absorbance.
